# Ambulantes Langzeit-Video-EEG als neuer diagnostischer Ansatz in Deutschland: Ergebnisse einer Machbarkeitsstudie

**DOI:** 10.1007/s00115-022-01412-0

**Published:** 2022-11-21

**Authors:** Christian Meisel, Martin Holtkamp, Simon Vock

**Affiliations:** 1 AG Computational Neurology, Department of Neurology, Charité – Universitätsmedizin Berlin, Charitéplatz 1, 10117 Berlin, Deutschland; 2 Berlin Institute of Health, Berlin, Deutschland; 3 Bernstein Center for Computational Neuroscience, Berlin, Deutschland; 4 Center for Stroke Research Berlin, Berlin, Deutschland; 5 NeuroCure Cluster of Excellence, Charité – Universitätsmedizin Berlin, Berlin, Deutschland; 6 Epilepsy-Center Berlin-Brandenburg, Institute for Diagnostics of Epilepsy, Berlin, Deutschland; 7 Department of Neurology, Charité – Universitätsmedizin Berlin, Berlin, Deutschland

**Keywords:** Epilepsie, Langzeit-Video-EEG, Ambulantes Monitoring, Epilepsy, Long-term video EEG, Outpatient monitoring

## Abstract

Das Langzeit-Video-EEG als Goldstandarddiagnostik bei Epilepsie und anderen Anfallsleiden ist in Deutschland derzeit nur in wenigen spezialisierten Zentren zur stationären Überwachung verfügbar. Diese begrenzten Überwachungskapazitäten und der damit verbundene hohe Zeit- und Arbeitsaufwand führen bundesweit zu einer erheblichen Wartezeit für diese wichtige Diagnostik. Neue, tragbare Sensortechnologien und automatisierte Datenanalysemethoden schaffen Möglichkeiten für Video-EEG-Langzeituntersuchungen nach dem Goldstandard im ambulanten Bereich, die dazu beitragen können, diese Barriere zu überwinden. Hier berichten wir über die Ergebnisse einer Machbarkeitsstudie an einem Zentrum, in der das ambulante Langzeit-Video-EEG (ALVEEG) als diagnostischer Weg in Deutschland eingeführt wurde. In dem neuen Diagnostikpfad soll der Einsatz innovativer, tragbarer Video-EEG-Monitoringsysteme zusammen mit einer durch künstliche Intelligenz unterstützten Datenanalyse den von Anfallsleiden betroffenen Patienten einen schnelleren, effizienteren und sektorübergreifenden Zugang zu einer Goldstandarddiagnostik in der häuslichen Umgebung ermöglichen. Die Diagnostik wurde von Patienten und Klinikern gut angenommen und könnte eine ergänzende Option zur stationären Überwachung darstellen, um die derzeitigen Engpässe in Diagnostik und Versorgung zu beseitigen.

**Zusatzmaterial online:**

Die Onlineversion dieses Beitrags (10.1007/s00115-022-01412-0) enthält eine zusätzliche Tabelle zu den Impedanzen der einzelnen Patienten.

## Abstract (en)

In Germany, long-term video EEG as the gold standard for the diagnostics of epilepsy and other seizure disorders, is currently only available for inpatient monitoring in a limited number of specialized centers. These limited monitoring capacities and the large amount of associated time and work resources lead to a significant waiting time for this important diagnostic procedure nationwide. New portable sensor technology and automated data analysis methods are creating opportunities for gold standard long-term video EEG assessments in outpatient settings, which may help to resolve this barrier. Here, we report the results of a single-center feasibility study by implementing outpatient long-term video EEG (ALVEEG) as a diagnostic pathway in Germany. In the new diagnostic pathway, the use of innovative, portable video EEG monitoring systems along with artificial intelligence-assisted data analysis are intended to provide those patients affected by seizure disorders with a more rapid, efficient, and cross-sectoral access to gold standard diagnostics in the home environment. The diagnostics were well accepted by patients and clinicians and may represent a complementary option to inpatient monitoring to eliminate current bottlenecks in diagnostics and care.

## Hintergrund und Fragestellung

Etwa 5 % der Bevölkerung erleiden einmal im Leben einen epileptischen Anfall, bis zu 1 % entwickelt eine Epilepsie. Damit gehören Epilepsien zu den häufigsten chronischen Erkrankungen des zentralen Nervensystems [1, 2]. Hinzu kommt eine hohe Zahl von Anfallsleiden anderer Ursache, bspw. synkopaler oder psychogener Genese, die differenzialdiagnostisch zugeordnet werden müssen [3]. Das Fehlen einer frühzeitigen und korrekten Diagnosespezifizierung kann bei einer unzureichend behandelten Epilepsie zu weiter auftretenden Anfällen, erheblichen Verletzungen und im schlimmsten Fall zum Tod führen („sudden unexpected death in epilepsy“, SUDEP), Letzteres gerade bei schlafgebundenen, tonisch-klonischen Anfällen [4]. Umgekehrt wird davon ausgegangen, dass bei ca. einem Viertel der mit Epilepsie diagnostizierten Patienten letztlich keine Epilepsie vorliegt [5–7]. Eine falsch-positiv diagnostizierte Epilepsie kann jahrelange Fehltherapien mit erheblichen Nebenwirkungen bedeuten und gravierende sozialmedizinische Auswirkungen haben. Insofern kommt dem zeitgerechten Zugang zu einer adäquaten Diagnostik eine herausragende Bedeutung zu [10].

Als Goldstandard in der Epilepsiediagnostik gilt ein stationäres, mehrtägiges Video-EEG-Monitoring [3, 8, 9]. In Deutschland ist diese Diagnostik jedoch nur begrenzt stationär verfügbar (ca. 200 Video-EEG-Monitoringplätze in Epilepsiezentren, Stand 2017 [5]) und wird durch die Nutzung vorhandener Kapazitäten für prächirurgische Beurteilungen und fehlendes Fachpersonal für das sehr zeitaufwendige Auswerten der großen Datenmengen weiter verschärft. Zur Lösung dieser Probleme wurde in anderen Ländern das ambulante Langzeit-Video-EEG (ALVEEG) als Versorgungsform eingeführt [11, 12]. Es besteht die Hoffnung, dass sich durch die ambulante Langzeit-Video-EEG-Diagnostik eine Reihe positiver Versorgungseffekte ergeben, darunter eine frühzeitigere und genauere Diagnose und Therapieanpassung nach dem Goldstandard, Vermeidung von Unter- und Fehlbehandlungen und damit verbundener gesundheitlicher Risiken wie Stürze und Tod (z. B. durch SUDEP) sowie Reduktion psychosozialer Konsequenzen für den Patienten. Neben einer Verbesserung der Versorgungseffizienz und Schonung von Ressourcen im stationären Bereich könnte eine ambulante Diagnostik bei dem Patienten zuhause auch die dort u. U. vorkommenden Anfallsauslösefaktoren besser abbilden. Mit der zunehmenden Verfügbarkeit innovativer, tragbarer, durch künstliche Intelligenz (KI) unterstützte Video-EEG-Diagnostik ergibt sich somit auch in Deutschland die Möglichkeit einer ambulanten Goldstandardepilepsiediagnostik im häuslichen Umfeld.

Die hier vorgestellte Studie hatte zum Ziel, die Machbarkeit ambulanter Langzeit-Video-EEG in Deutschland zu untersuchen, für die es bisher keinen definierten Diagnostikpfad gibt. Dabei wurden neben der Beurteilung der benötigten strukturellen Voraussetzungen zur Implementierung dieser Diagnostik auch die Einschätzungen und Bewertungen von Behandlern und Patienten berücksichtigt.

## Studiendesign und Untersuchungsmethoden

### Machbarkeitsstudie

Ziel der Machbarkeitsstudie war die Beurteilung von Durchführbarkeit, Verträglichkeit und Patientenakzeptanz ambulanter Langzeit-Video-EEG für Menschen mit Anfallserkrankungen (ALVEEG). Mobile Video-EEG-Monitoringsysteme werden von verschiedenen Herstellern angeboten [15, 16, 19]. Für die hier durchgeführte Studie wurde das System der Firma Seer genutzt, welches CE-zertifiziert ist und die gleichzeitige Durchführung von EEG (21 Kanäle, 10–20-System), EKG (2 Kanäle) und Videographie über mehrere Tage beim Patienten zuhause erlaubt. Das Diagnostiksystem, der Ablauf der Studie und die Datenflüsse wurden hinsichtlich der Einhaltung von Datenschutzvorgaben im Vorfeld durch das Clinical Trial Office der Charité evaluiert. In diesem Zusammenhang wurde auch bestätigt, dass Daten nur auf DSGVO-konformen Plattformen innerhalb Deutschlands verarbeitet werden dürfen, entsprechende Anpassungen des Diagnostiksystems wurden daraufhin vorgenommen. Die Studie erhielt ein positives Ethikvotum durch die Ethikkommission der Charité – Universitätsmedizin Berlin und eine schriftliche Einwilligung wurde von allen Patienten nach Aufklärung über den Studieninhalt eingeholt.

### Ablauf

Potenzielle Patienten wurden prospektiv zwischen November 2021 und Februar 2022 über die Hochschulambulanzen der Klinik für Neurologie der Charité identifiziert. Wir folgten dem folgenden Protokoll:

1. Potenzielle Patienten für das ALVEEG wurden zunächst vom medizinischen Personal in den Epilepsieambulanzen der Charité auf ihre Eignung hin überprüft. Wir wählten insbesondere Patienten mit bekannter Epilepsie aus, bei denen eine Indikation 1–4 der OPS 1–210 zum diagnostischen Langzeit-Video-EEG-Monitoring (LVEM) vorlag: Evaluation der Art der Anfälle, Evaluation des Epilepsiesyndroms, Evaluation bei therapierefraktärer Epilepsie, Evaluation zur weiteren Therapieplanung. Patienten mit Indikation für ein prächirurgisches Monitoring wurden ausgeschlossen. Neben der Auswahl entsprechend der OPS-Kriterien wählten wir primär Patienten aus, von denen wir annahmen, dass sie mit der Technik gut zurechtkommen würden. Nach Feststellung der Indikation für ein LVEM wurden ein Ambulanztermin mit dem Patienten vereinbart und ein Informationsblatt im Vorfeld zugesandt.
2. Bei dem Ambulanztermin erhielt der Patient eine Einweisung in den Umgang mit der Technik und über den Ablauf der Untersuchung. Anschließend wurden ihm die Elektroden an Kopf (EEG, 21 Kanäle, 10–20-System) und Brustkorb (EKG, 2 Kanäle) angelegt. Beim EEG kamen wasserbasierte, mit Gel fixierte Elektroden zum Einsatz. Die Elektroden waren mit einem tragbaren, batteriebetriebenen EEG/EKG-Gerät verbunden, dessen Daten an einen in einem Rollkoffer integrierten Datenrekorder übertragen wurden. An diesem Rollkoffer befand sich auch eine Nachtsichtkamera. Die gesamte Ausrüstung verteilte sich auf diesen Rollkoffer sowie eine weitere Tasche und konnte von den Patienten gut bewegt werden. Der Transport in die Häuslichkeit erfolgte den individuellen Vorstellungen der Patienten entsprechend mit den öffentlichen Verkehrsmitteln oder durch Abholung mit dem Auto.
3. Während des Monitorings beim Patienten zuhause wurde der Rollkoffer mit ausgezogener Kamerastange in dem Raum platziert, in dem der Patient die meiste Zeit verbrachte. Ein magnetischer Anschluss an der EEG-Elektrodeneinheit am Kopf erlaubte es dem Patienten, das EEG-EKG-Gerät kurzfristig abzulegen, beispielsweise um zu duschen. Die Qualität der Datenaufzeichnung und der Impedanzwerte wurden mindestens einmal täglich durch das Studienpersonal telemedizinisch kontrolliert. Das verwendete Seer-System erlaubte eine stichprobenartige Messung und Darstellung der Impedanzwerte in Echtzeit, sofern der Patient mit dem System und der Cloud verbunden ist. Bei Bedarf wurden die Patienten telefonisch unterstützt, Elektroden zu refixieren. Die Patienten wurden gebeten, über eine App oder ein Anfallstagebuch ihre Anfälle und andere relevante Ereignisse zu erfassen. Diese Angaben wurden später mit den Video-EEG-EKG-Daten in der Onlineplattform synchronisiert und waren bei der Auswertung für das klinische Personal sichtbar. Während des Monitorings wurden die Video-EEG-EKG-Daten automatisch lokal in dem Gerät beim Patienten zuhause gespeichert. Nach Ende des 3‑ bis 7‑tägigen Monitorings konnte der Patient die mit einem wasserbasierten Gel fixierten EEG-Elektroden eigenständig abwaschen, er gab die Ausrüstung dann in der Ambulanz zurück.
4. In der Ambulanz wurden die gesammelten Daten in eine DSGVO-konforme Onlineplattform geladen und standen dort zur Einsicht und Befundung zur Verfügung. Dabei konnte auch ein validierter und in der realen Versorgung erprobter Algorithmus genutzt werden [15], welcher für epilepsietypische Aktivität verdächtige iktale und interiktale Datensegmente niederschwellig markierte (hohe Sensitivität, niedrige Spezifität) und so die Auswertung und Befundung u. U. unterstützen und beschleunigen konnte.
5. Der Befundbericht wurde dem primär zuständigen Neurologen zugestellt, der diesen in einem gesonderten Termin mit dem Patienten besprach und gemeinsam mit dem Patienten sich ggf. daraus ergebende Therapieschritte umsetzte. Über einen Webbrowser konnte auch der zuweisende Arzt von überall auf die Video-EEG-EKG-Daten und Befunde in der Onlineplattform zugreifen.

### Endpunkte

Ein Ziel der Machbarkeitsstudie war die Erfassung von Akzeptanz von ALVEEG bei den Patienten und Klinikern, welche durch Fragebögen systematisch erfasst wurden. Der Fragebogen für Patienten umfasste folgende Punkte (bewertet jeweils mit Punkten 1–5; 5 am besten):

- Wie würden Sie den Gesamtkomfort des Systems bewerten?
- Wie würden Sie den Komfort während des Schlafens und Duschens bewerten?
- Wie würden Sie insgesamt die Bedienbarkeit des Systems bewerten?
- Wie bewerten Sie die Einschränkungen durch das System in Ihrem täglichen Leben?
- Würden Sie generell diese ambulante Diagnostik der sonst üblichen stationären Langzeit-Video-EEG-Diagnostik bevorzugen?
- Würden Sie diese Diagnostik weiterempfehlen?

Des Weiteren bestand die Möglichkeit für weitere Anmerkungen in einem Kommentarfeld.

Da es sich bei ALVEEG um Veränderungen in der klinischen Praxis handelt, haben wir auch die beteiligten Fachärzte und MTAs mit Fragebögen befragt, um herauszufinden, wie ALVEEG wahrgenommen wird. Der Fragebogen umfasste die folgenden Punkte:

- Zurechtkommen mit der Technik? (1–5 bewertet nach Punkten; 5 am besten)
- Dauer Anbringen der Elektroden?
- Dauer Anbringen des Systems insgesamt?
- Zwischenfälle oder Komplikationen?
- Wie oft bestand Kontakt mit dem Patienten während des Monitorings?

Auch hier bestand die Möglichkeit für weitere Anmerkungen in einem Kommentarfeld. Darüber hinaus erfassten wir folgende Parameter: Dauer der geplanten Aufzeichnung, Dauer der tatsächlich durchgeführten Aufzeichnung, benötigter Zeitaufwand der MTA insgesamt, Zeitaufwand der Befundung durch Neurologen, ob und welche Art relevante klinische und elektrographische Ereignisse während der Aufzeichnung erfasst wurden.

## Ergebnisse

Nach positiv beschiedener Evaluation hinsichtlich Datenschutzkonzept und Ethik durch die entsprechenden Gremien an der Charité rekrutierten wir 5 Patienten für die Studie (mittleres Alter 36 Jahre, Tab. 1). Keiner der letztlich für die Diagnostik ausgewählten Patienten sagte die Untersuchung ab. Die Patienten hatten alle eine bekannte Epilepsie. Die an das diagnostische Langzeit-Video-EEG gestellten Fragen betrafen die Quantifizierung der Anfälle, die Art der Anfälle und die Frage nach schlafgebundenen Anfällen. Die Patienten wurden über die Hochschulambulanz rekrutiert und über die Studie informiert. Nach Einwilligung erfolgten zu einem gesonderten Termin die Anlage der EEG- und EKG-Elektroden und die Einweisung in die Technik durch eine Studien-MTA. Der Arbeitsaufwand der Studien-MTA über die gesamte Zeit des Monitorings schwankte zwischen 2 und 4 h (Mittelwert 3 h, Tab. 1). Die Studien-MTA gab an, mit dem System und der Technik gut zurechtzukommen (Mittelwert 4,8).

**Table 1:** 

Patient #	Aufgenommene Anfälle	Interiktale Aktivität	Monitoringdauer (Tage)	Benötigte Zeit MTA (Stunden insgesamt)	Dauer Befundung (Stunden)
1	Nein	Ja	3	3	2
2	Nein	Ja	6	2	3,5
3	18 hyperkinetische Anfälle	Ja	5	4	4
4	Nein	Nein	3	3,5	2
5	1 bilateral tonisch-klonischer Anfall	Ja	6	2,5	3
*Mittelwert*	*–*	*–*	*4,6*	*3*	*2,9*

Die Dauer des Monitorings bei dem Patienten zu Hause variierte zwischen 3 und 6 Tagen (Mittelwert 4,6 Tage, Tab. 1). Alle Monitorings konnten mit der geplanten Dauer durchgeführt werden. Wir kontrollierten jeden Tag mindestens einmal die Elektrodenimpedanzen, was mit dem System ferndiagnostisch möglich war. Wenn sich dabei ein Problem mit einer oder mehreren Elektroden zeigte, wurde der Patient telefonisch kontaktiert und je nach Bedarf eine Elektrodenpflege durchgeführt, bei der der Patient telefonisch unterstützt wurde. Insgesamt führten wir eine Elektrodenpflege bei 3 von 5 Patienten mindestens einmal durch. Bei den hier untersuchten Patienten konnte dadurch jede Elektrode wieder angebracht werden. Stichpunktartige Messungen der Medianwerte der Impedanzen für die Patienten 1 bis 5 erbrachten zu Beginn der Untersuchung Werte von 1, 0, 1, 3 und 1 kΩ, zum Ende der Untersuchung Werte von 3, 18, 6, 28 und 14 kΩ, wobei diese Werte auch tageszeit- und aktivitätsabhängigen Schwankungen unterlegen waren (Impedanzwerte in Supplementary Materials, Tabelle S1). Die Patienten berichteten von einer insgesamt guten Bedienbarkeit und einem guten Auskommen mit der Technik (Mittelwert 4,2), einem guten Komfort insgesamt (Mittelwert 3,8) und während des Schlafens bzw. Duschens (Mittelwert 3,6). Die Patienten gaben an, eine derartige ambulante Diagnostik gegenüber einer stationären zu bevorzugen (Mittelwert 4,4) und sahen diese Form der Diagnostik als empfehlenswert an (Mittelwert 4,4).

**Figure 1:**
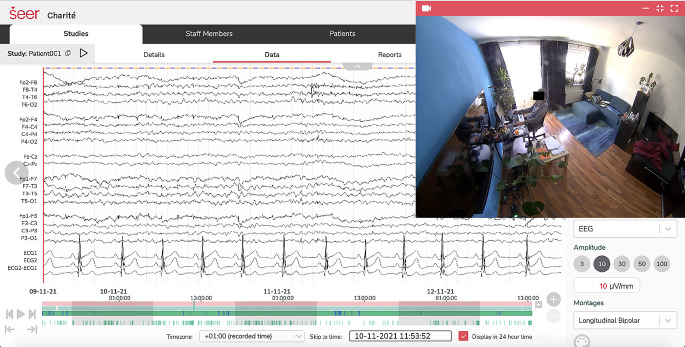

Die Befundung der Langzeit-Video-EEG-Daten erfolgte durch zwei Neurologen mit langjähriger Erfahrung in der Befundung von EEGs (CM, MH). Abb. 1 zeigt beispielhaft die Darstellung der in der Häuslichkeit des Patienten aufgenommenen Langzeit-Video-EEG-EKG-Daten. Epileptische Anfälle zeigten sich bei 2 von 5 Patienten (18 hyperkinetische Anfälle bei einem Patienten, ein bilateral tonisch-klonischer Anfall bei einem weiteren Patienten). Alle Anfälle ereigneten sich nachts aus dem Schlaf heraus, und keiner der Patienten gab retrospektiv an, während der Monitoringperiode Anfälle erlitten zu haben. Bei allen Anfällen war der Patient gut im Video sichtbar. Auch wurde die EEG- und EKG-Datenqualität als gut eingeschätzt. Es wurden zunächst die vom System markierten Abschnitte angesehen. Oftmals konnten diese Abschnitte schon die für die Befundung relevanten Fragestellungen beantworten. In einem nächsten Schritt wurden dann die Nächte anhand der EEG-Ableitungen sowie stichprobenartig auch über den Tag verteilte Datenabschnitte beurteilt. Dabei wurde bei der Beurteilung auch das Video hinzugezogen. Bei 4 von 5 Patienten wurde interiktale epilepsietypische Aktivität in Form scharfer Wellen und Spitzen dokumentiert. Das benutzte System lieferte die Möglichkeit einer automatisierten Markierung potenziell iktaler oder interiktaler EEG-Segmente, was von den Befundern als hilfreich und für die Befundung generell als beschleunigend empfunden wurde. Bei den hyperkinetischen Anfällen wurden die EEG-Anfallsmuster von diesem System nicht automatisch erkannt, was die Notwendigkeit einer weiterhin manuellen Kontrolle verdeutlicht. Komplett nicht auswertbar war das EEG in dieser Studie nur während der Zeit, wenn die Patienten das EEG-Aufnahmesystem von den Elektroden dekonnektiert hatten, z. B. um zu duschen. Nach Schätzung der befundenden Neurologen war dies in weniger als 5 % der Zeit der Fall. Ebenso waren die Patienten nach Abschätzung der befundenden Neurologen den ganz überwiegenden Anteil der Zeit vollständig im Video sichtbar und geschätzt weniger als 5 % der Zeit nicht oder nur teilweise im Video sichtbar. Die Dauer der Befundung durch die Neurologen variierte insgesamt zwischen 2 und 4 h (Mittelwert 2,9 h) pro Patient.

## Diskussion

Bislang ist ein mehrtägiges Video-EEG-Monitoring als Goldstandard in der Epilepsiediagnostik in Deutschland nur im stationären Bereich verfügbar. Ziel der vorliegenden Studie war die Untersuchung von Machbarkeit und Akzeptanz eines ambulanten Langzeit-Video-EEG (ALVEEG) für die Anwendung in Deutschland. Ein leichterer, zeitnaher Zugang zur Goldstandarddiagnostik durch ein ambulantes Angebot ist für Diagnosestellung (inkl. differenzialdiagnostischer Abgrenzung), syndromale Zuordnung einer Epilepsie und Therapiekontrolle mit Frage nach Art und Anzahl von Anfällen erstrebenswert. Die Patientenangaben zu Art und Anzahl von Anfällen sind bekanntermaßen oft nicht valide [20]. Auch in der aktuellen kleinen Stichprobe von Patienten zeigte sich, dass die nächtlichen Anfälle bei beiden Patienten mit Anfällen im Nachhinein nicht erinnerlich waren. Dies deckt sich mit Studien aus dem stationären Monitoring, sodass von einer hohen Dunkelziffer undokumentierter Anfälle auszugehen ist. Ein niederschwelligeres Angebot zum LVEM könnte diesbezüglich Abhilfe schaffen und einen Beitrag für eine realistischere Datenbasis für Diagnostik und Therapieoptimierung bei Patienten mit Epilepsie leisten. Perspektivisch kann eine solche Diagnostik in der Breite möglicherweise auch bei Fragen zur Einschätzung der Fahreignung, der individuellen Risikobewertung oder zur Stärkung der Gesundheitskompetenz für die Patienten sinnvoll sein.

Die neue Diagnostik wurde von den Patienten allgemein gut akzeptiert, wobei es sich in der vorliegenden Machbarkeitsstudie um ein selektiertes Patientenkollektiv handelt. So wurden von uns bislang keine Kinder, keine älteren Menschen und keine Menschen mit Intelligenzminderung mit dieser Diagnostik versehen. Studien aus anderen Ländern scheinen aber auch für diese Patientenkollektive einen Nutzen und eine hohe Akzeptanz für das ambulante Monitoring zu erkennen [11]. Studien mit einem größeren und breiteren Patientenkollektiv erscheinen zur Beantwortung dieser Fragen auch in Deutschland sinnvoll. Basierend auf den Ergebnissen der hier untersuchten Patientenkohorte erscheint ALVEEG mindestens für bestimmte Patienten als eine zum stationären Monitoring komplementäre gute Alternative. In der vorliegenden Studie dokumentierte sich auch nach Einschätzung der befundenden Neurologen eine ausreichende EEG- und Videodatenqualität, wie sie für eine epileptologische Diagnostik benötigt wird. Bei den hier untersuchten Patienten konnte jede Elektrode wieder angebracht werden, was natürlich nicht ausschließt, dass dies dennoch passieren kann. Zudem waren die Patienten die ganz überwiegende Zeit gut und vollständig auf dem Video zu erkennen und die EEG-Qualität, soweit beurteilbar, vergleichbar dem stationären Monitoring. Eine Einschränkung des ambulanten Ansatzes im Vergleich zu dem stationären Setting ist, dass keine iktale, orientierende neuropsychologische Testung der Patienten durch eine MTA – wie in einigen, aber nicht allen Epilepsiezentren möglich – durchgeführt werden kann. Zudem ist eine Reduktion der Dosis der antikonvulsiven Medikamente im ambulanten Setting in der Regel nicht möglich ist, weil keine Überwachung durch MTAs im Fall von Anfallsserien oder Status epilepticus vorhanden ist. Zukünftige Studien mit dem Ziel eines direkten Vergleichs zwischen stationärem und ambulantem Langzeit-Video-EEG hinsichtlich dezidierter Endpunkte, wie Proportion der gelösten klinischen Fragestellungen, erscheinen daher sinnvoll. Ebenso erscheinen eine detaillierte Validierung der Performanz des verwendeten KI-Systems sowie dessen Vergleich mit anderen Algorithmen in zukünftigen Studien sinnvoll. Diese Fragestellung wäre über das aktuelle Studienziel, das primär die Beurteilung von Machbarkeit und Einsetzbarkeit von ALVEEG beinhaltete, hinausgegangen. Für eine Translation in die breitere Versorgung wird es zudem noch Fragen zur Erstattung durch die Kostenträger zu klären geben. Bislang gibt es in Deutschland noch keine entsprechenden kassenärztlichen Vergütungsmechanismen.

Das ambulante Langzeit-Video-EEG steht beispielhaft für die zunehmenden Möglichkeiten und Chancen der multimodalen und kontinuierlichen Erfassung longitudinaler Patientendaten durch Digitalisierung, neue Sensorik und tragbare Technologien im ambulanten Setting. Die Komplexität und Menge an Informationen dieser Big Data stellt gleichzeitig eine große Herausforderung für Datenanalyse und -qualitätssicherung bei der Translation in die klinische Versorgung dar. Eine traditionelle, ausschließlich manuelle Befundung der Daten würde diesen neuen Ansatz jedoch schon aufgrund der erforderlichen personellen Ressourcen, welche für die Auswertung eines mehrtägigen Video-EEG benötigt werden, in der Breite verhindern. Um aus den Daten die entscheidenden Informationen effizient und valide zu extrahieren, bedarf es eines neuen interdisziplinären Ansatzes, welcher klinisch-neurologische Expertise mit Datenwissenschaft und verwandten Disziplinen, wie Informatik, vernetzt und, wie hier, eine KI-unterstützte hybride Befundung gezielt einsetzt. Methoden des maschinellen Lernens angewandt auf Wearables [13, 14, 17], EEG oder Videodaten [18] bieten hier Chancen eines effizienteren und objektiveren Monitorings und Dokumentation von Anfallsereignissen [20].

Die hiesige Machbarkeitsstudie zeigt, dass sich derartige ambulante Monitoringansätze auch strukturell in Deutschland umsetzen lassen. Mit dem weiteren Ausbau der digitalen Medizin, der Telemedizin und der ambulanten Langzeitüberwachung werden rechnergestützte Ansätze der Neurologie in Zukunft weiter an Relevanz gewinnen. Dies erfordert auch, dass Datenwissenschaftler sich mit klinischen Aspekten befassen müssen, und dass Ärzte sich mit datenwissenschaftlichen Aspekten vertraut machen, um in Zukunft die beste Diagnostik für ihre Patienten auszuwählen und anzuwenden. Zusammen bietet sich damit die Chance für neue und effizientere Langzeitdiagnostik im ambulanten Setting.

## Fazit für die Praxis

Durch neue, tragbare Sensorik und teilautomatisierte Datenanalysemethoden ergeben sich Chancen einer ambulanten Langzeit-Video-EEG-Diagnostik nach dem Goldstandard. Die vorliegende Machbarkeitsstudie zeigt die prinzipielle Umsetzbarkeit einer solchen Diagnostik in Deutschland und deren gute Akzeptanz durch Patienten und Kliniker. Das ambulante Langzeit-Video-EEG stellt damit potenziell eine komplementäre Ergänzung zum stationären Monitoring dar, um aktuelle Versorgungsengpässe zu beseitigen.

## Supplementary Information




