# Supplementary material for: Outpatient long-term video EEG as new diagnostic approach in Germany: results of a feasibility study
Source: Nervenarzt. 2022 Nov 21;94(6):519–24. [Article in German] doi: 10.1007/s00115-022-01412-0 (PMC10256646; doi:10.1007/s00115-022-01412-0)
Supplement: Supplementary file 1 [file 115_2022_1412_MOESM1_ESM.docx]

# Ambulantes Langzeit-Video-EEG als neuer diagnostischer Ansatz in Deutschland: Ergebnisse einer Machbarkeitsstudie

# Supplementary Material

| Patient # | Impedanz in kΩ; Medianwert, Mittelwert, Minimum, Maximum | | | | | |
| --- | --- | --- | --- | --- | --- | --- |
|  | Tag 1 | Tag 2 | Tag 3 | Tag 4 | Tag 5 | Tag 6 |
| 1 | 1, 1, 0, 5 | 1, 7, 0, 78 | 3, 38, 1, 251 |  |  |  |
| 2 | 0, 1, 0, 5 | 1, 2, 0, 18 | 1, 6, 0, 75 | 3, 18, 1, 168 | nicht gespeichert | 18, 65, 0, 151 |
| 3 | 1, 1, 0, 3 | 2, 3, 1, 8 | 2, 4, 1, 12 | 6, 24, 4, 140 | 6, 18, 2, 194 |  |
| 4 | 3, 11, 1, 83 | 19, 47, 1, 178 | 28, 43, 1, 171 |  |  |  |
| 5 | 1, 2, 0, 7 | 1, 2, 0, 6 | 5, 20, 3, 142 | nicht gespeichert | nicht gespeichert | 14, 66, 2, 87 |

Tabelle S1: Übersicht zu den Impedanzen der einzelnen Patienten.
